# Overwintering and Resident Birds in Qatar: Explorations With DNA Barcoding

**DOI:** 10.1002/ece3.71817

**Published:** 2025-07-15

**Authors:** Emily Rebecca Alison Cramer, Kuei‐Chiu Chen, Arild Johnsen

**Affiliations:** ^1^ Marine Sciences, Tjärnö Marine Laboratory University of Gothenburg Strömstad Sweden; ^2^ Department of Premedical Education Weill Cornell Medicine‐Qatar Education City Qatar; ^3^ Natural History Museum University of Oslo Oslo Norway

**Keywords:** avifauna, cytochrome *c* oxidase I (COI), DNA barcoding, Middle East, migration

## Abstract

Genetic research is unevenly distributed across the globe, with most research done in temperate zones. To better understand the birdlife in an under‐represented, arid subtropical country, Qatar, we blood sampled birds and salvaged tissue from dead birds, then sequenced a mitochondrial marker (cytochrome *c* oxidase subunit I, COI, the “DNA barcoding” gene). We chose the DNA barcoding gene because it has previously proven useful for preliminary explorations of evolutionary history. We obtained DNA barcodes for 115 birds (34 species, 12 orders). Our data suggest that the existing DNA barcode reference library, built largely from sampling in the Americas, Europe, and east Asia, is generally sufficient for species identification in Qatar. Based on DNA barcode similarity, Qatar provides overwintering habitat to some species with apparent strong migratory connectivity and others with weaker migratory connectivity. Among locally breeding species, we found no evidence of hybridization between House Sparrows *Passer domesticus* (*n* = 16 males) and Spanish Sparrows *P. hispaniolensis* (*n* = 14 males), breeding simultaneously and in the same habitats, although in other locations of range overlap, habitat segregation and timing of breeding are hypothesized to be the primary reproductive barrier between them. Our results highlight the benefits of expanding the geographic range of genetic and ecological research.

## Graphical abstract

We obtained DNA barcodes for 115 birds (34 species, 12 orders) in Qatar, a country with no previous barcode sequence data for wild birds. We provide insights on locally breeding species and overwintering migrants.

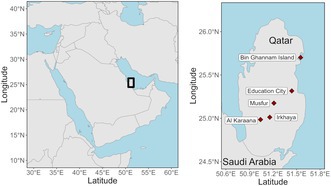

## 1 Introduction

Research in ecology and evolution has been geographically biased, with most work conducted in the temperate zone (Culumber et al. 2019; Kartzinel et al. 2025). Correcting that bias is important for developing more generalizable evolutionary and ecological theory (Zuk 2016; Culumber et al. 2019), and it can also have important conservation implications (Kartzinel et al. 2025). For example, many migratory birds breed in the temperate zone and overwinter in the tropics, so it is important for conservation efforts to account for birds' distribution during all parts of their annual cycle (DeLuca et al. 2023).

One region where few genetic studies on wildlife, and specifically birds, have been conducted is the subtropical arid Arabian peninsula (but see some recent exceptions, e.g., Campbell et al. 2019; Draidia et al. 2022; Zogaris and Kallimanis 2016). This region lies on the East Asian/East African flyway, such that it can provide important stop‐over and overwintering locations for migrating birds. Habitat availability in Qatar can change rapidly due to urban and agricultural development that can result in the novel addition of freshwater and mesic habitats, including cropland and wetland areas (Draidia et al. 2022; Aloui et al. 2023). To the best of our knowledge, no genetic data were previously collected on wild birds in this country (e.g., Figure S1). Genetic data are an important supplement to phenotypic data, because genetic and phenotypic differentiation are not always parallel, resulting in phenotypically highly similar species with substantially differentiated genomes (“cryptic species” sensu Struck et al. 2018) and, conversely, genomically highly similar animals with strikingly different phenotypes (e.g., Toews et al. 2016). A useful tool for a first exploration of genetic differentiation is the mitochondrial cytochrome *c* oxidase subunit I (COI) gene, which generally shows low intraspecific differentiation and high interspecific differentiation (the barcode gap, Hebert et al. 2003, 2004). Exceptions to this rule often reflect complex and interesting evolutionary histories (Johnsen et al. 2010; Hogner et al. 2012; Barreira et al. 2016). The availability of sequence data from many individuals, due to DNA barcoding initiatives, increases its utility for exploring evolutionary history (Ratnasingham and Hebert 2007).

Here, we ringed and blood sampled birds and analyzed the DNA barcode of these individuals, as well as salvaged dead birds, in Qatar, a country on the eastern side of the Arabian Peninsula. We explore the implications of these new data for understanding Qatar's avian biodiversity, and we describe observations relevant for overwintering ecology.

## 2 Materials and Methods

### 2.1 Field Methods

**FIGURE 1:**
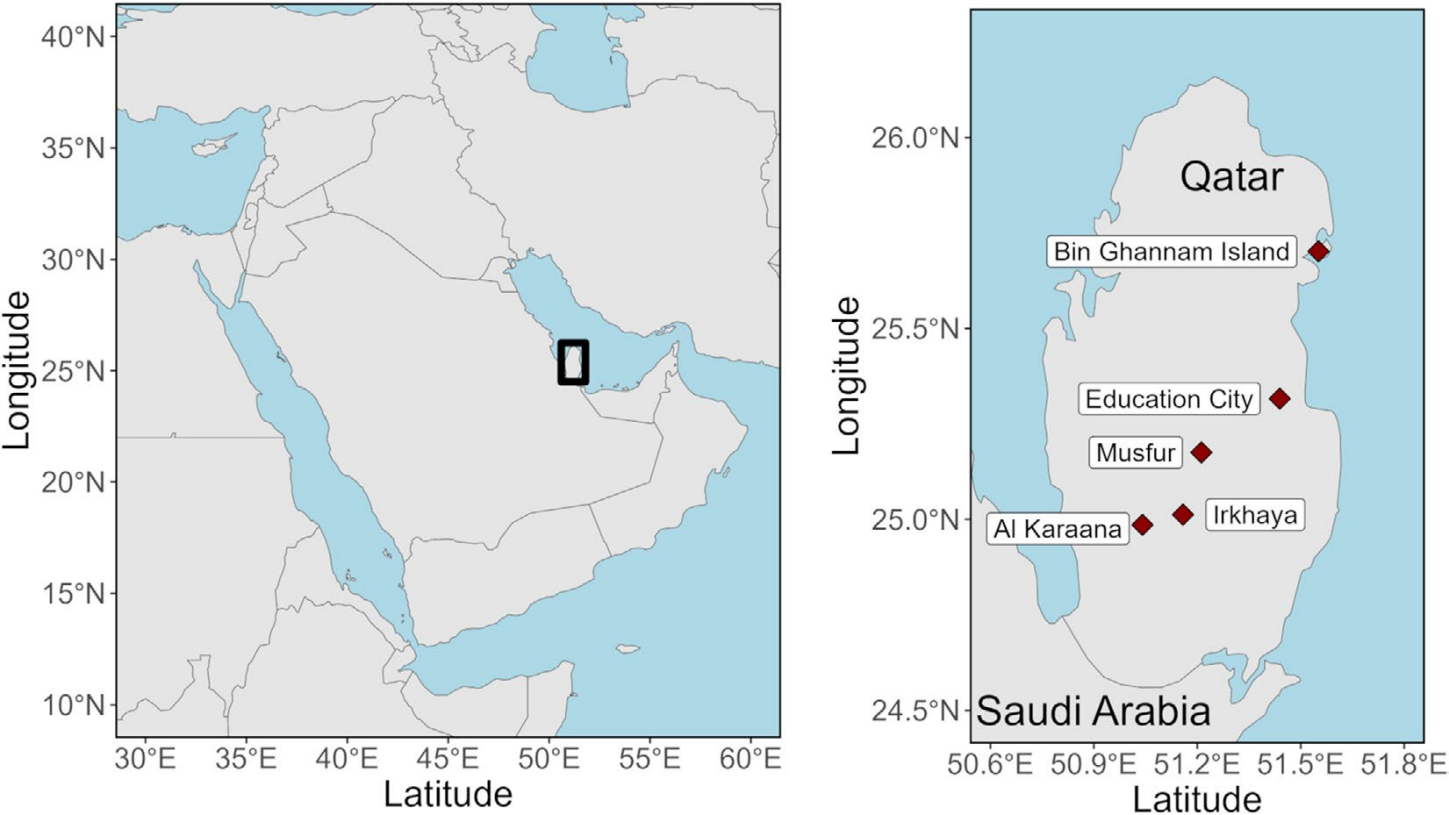
Map showing locations for primary field sites (right) and larger image of the region including Qatar (left).

Birds were captured using mistnets (passively or with playback) at several field sites in Qatar (Figure 1); in addition, samples were taken opportunistically from birds found dead. We sampled from animals found dead between January 2019 and March 2023. Mistnetting was conducted regularly from 24 January 2020 to 20 March 2020, as well as on a few days in 2019 and 2022 (details in Supporting Information). We typically used one 12‐m, one 9‐m, and one 6‐m net, and netted from dawn until late morning. Playback was primarily of migratory European‐breeding species, with particular emphasis on *Luscinia svecica*. Field sites were chosen based on a combination of accessibility and suitability for mistnetting, and they represent only a subset of the habitats present in Qatar (Figure S2); a few attempts at netting in the open desert scrubland were predictably unsuccessful due to wind and sun exposure. In addition to *Luscinia svecica*, specific effort was made to capture *Passer domesticus* and *Passer hispaniolensis* in light of our research interest in these species (e.g., Cramer et al. 2014, 2016). Thus, capture success does not reflect species prevalence within the habitats where we worked, nor in Qatar more generally.

Upon capture, a uniquely numbered metal ring was applied (provided by the Stavanger Museum, Norway; ringing data were reported to them, as Qatar does not have a bird ringing agency). Birds were assessed for molt, fat accumulation in the furcula, and brood patch or cloacal protuberance. Standard morphological measurements were taken (detailed in the Supporting Information). Blood (generally 15–20 μL; up to approximately 40 μL from heavier resident species) was taken by venipuncture of the brachial vein and stored in 70% ethanol until analysis. Birds were released immediately after sample collection at the location of capture. For the dead specimens collected opportunistically, muscle tissues of approximately 50 mg were collected and preserved in 70% ethanol. One hundred thirteen of 116 individuals were photographed. Samples from most species (including all live birds captured through 2020, except *Passer domesticus* and *Passer hispaniolensis*, which were instead reserved for other projects) were accessioned to the DNA bank of the Natural History Museum of the University of Oslo (accessions NHMO‐BI‐106481 to 106543); photos, measurements, and GPS locations of capture were also included with these accessions. Photos are also included in the BOLD dataset associated with this paper (DOI: https://doi.org/10.5883/DS‐QAVES), and morphological measurements are in the Data Dryad dataset https://doi.org/10.5061/dryad.kh18932kh.

Species identity was determined using two field guides; more formal European ringing resources were not available to us during the fieldwork period. In cases where genetic data conflicted with field identities (detailed below), we evaluated our field decisions based on the morphometric and photographic data, and we updated the species identity listed in the BOLD database where relevant. We follow taxonomy from the International Ornithological Committee (Gill et al. 2024).

### 2.2 Ethics Statement

All work was approved by the Weill Cornell Medicine‐Qatar Institutional Animal Care and Use Committee (2014‐0002) and conformed with local legislation of the Ministry of Municipality & Environment (MME2018‐006).

### 2.3 Lab Methods and Sequence Analysis

DNA was extracted from blood samples using standard spin‐column extraction kits (e.g., Omega EZNA kits and Qiagen DNeasy Blood and Tissue Cat. No. 69506) following the manufacturer's guidelines. The COI region was amplified via polymerase chain reaction (PCR) using the following primer pairs: BirdF1, BirdR2/CO1BirdR2 (Hebert et al. 2004), ExtF and ExtR (Johnsen et al. 2010), PasserF1 and PasserR1 (Lohman et al. 2009), and C_VF1LFt1 and C_VR1LRt1 (Ivanova et al. 2007). Details on which primers were used for which specimens and primer sequences are available on the BOLD database (specimen data in the project QAVES). Amplified DNA was then Sanger‐sequenced using the same primers (or with the M13F/M13R sequencing primer, for C_VF1LFt1/C_VR1LRt1 amplicons). Lab work was done for some samples at Weill Cornell Medicine‐Qatar (WCMQ), with sequencing by WCMQ's core facility. Most other samples were analyzed at the University of Oslo Natural History Museum (NHM), with sequencing by Macrogen Europe. A few *Passer* sparrow samples were extracted at the University of Nottingham but amplified at the NHM and sequenced at Macrogen. In general, we attempted to sequence amplicons in both the forward and reverse directions, but due to logistical constraints, we occasionally were only able to sequence in one direction. One specimen, with traits appearing like *Curruca communis* and/or *C. curruca*, did not return adequate sequences after repeated attempts with several primer pairs, which could reflect a pseudogene (i.e., a nuclear copy of the mitochondrial gene; Sorenson and Quinn 1998). Another specimen, *Falco tinnunculus*, was tested with only one primer pair due to logistical constraints and returned interpretable but lower‐quality sequence.

Where available, forward and reverse records were aligned, and base calls were edited manually in Mega 11.0.13 (Kumar et al. 2018) or Geneious Prime 2022.2.2 (Biomatters Ltd). These sequences and raw trace files were uploaded to BOLD to the project QAVES.

### 2.4 Analysis

To assess the agreement between genetic and phenotypic information, we submitted each DNA barcode sequence as a query on the BOLD automatic ID engine v4 with the Species‐Level Barcodes Records option, and we downloaded the identification trees in cases where the sequence could not automatically be identified to species level. For species where we sequenced more than one individual, we examined within‐species diversity within the Qatari samples using BOLD's distance tool (with Kimura 2 parameter). To evaluate the presence of the barcoding gap, we further used BOLD's distance tool to examine the between‐species, within‐genus, and within‐family distances, using all the individuals we sampled. We used the BOLD aligner with default settings, without excluding sequences based on length, in both sets of distance analyses.

Maps were created and data were managed in R (R Development Core Team 2012) using the packages tidyverse (Wickham et al. 2019), ggplot2 (Wickham 2016), sf (Pebesma 2018; Pebesma and Bivand 2023), rnaturalearth (Massicotte and South 2023), rnaturalearthdata (South 2017), and rnaturalearthhires (South et al. 2024).

## 3 Results

In total, obtained sequence for 115 individuals (33 species encompassing 12 taxonomic orders; Table 1). We first describe agreement between genetic and phenotypic species identification, as some initial field identifications were re‐assessed after we examined the genetic data. We then describe additional results relevant to the ecology and evolution of birds in Qatar.

**TABLE 1:** Number of individuals for each species with COI sequenced. Identifications here are the final identification based on field and genetic information.

Order	Family	Species	Common name	*N*
Galliformes	Phasianidae	*Ortygornis pondicerianus*	Gray Francolin	1
Columbiformes	Columbidae	*Streptopelia decaocto*	Eurasian Collared Dove	1
		*Spilopelia senegalensis*	Laughing Dove	9
Gruiformes	Rallidae	*Gallinula chloropus*	Common Moorhen	1
		*Fulica atra*	Eurasian Coot	1
Podicipediformes	Podicipedidae	*Tachybaptus ruficollis*	Little Grebe	1
		*Podiceps cristatus*	Great Crested Grebe	1
Phoenicopteriformes	Phoenicopteridae	*Phoenicopterus roseus*	Greater Flamingo	1
Charadriiformes	Recurvirostridae	*Himantopus himantopus*	Black‐winged Stilt	1
	Scolopacidae	*Arenaria interpres*	Ruddy Turnstone	1
	Laridae	*Larus fuscus*	Lesser Black‐backed Gull	1
		*Larus cachinnans*	Caspian Gull	1
Suliformes	Phalacrocoracidae	*Phalacrocorax carbo*	Great Cormorant	1
Accipitriformes	Pandionidae	*Pandion haliaetus*	Osprey	1
	Accipitridae	*Circus aeruginosus*	Western Marsh Harrier	1
Strigiformes	Strigidae	*Otus scops*	Eurasian Scops Owl	1
		*Bubo ascalaphus*	Pharaoh Eagle‐Owl	1
Coraciiformes	Meropidae	*Merops persicus*	Blue‐cheeked Bee‐eater	1
Falconiformes	Falconidae	*Falco tinnunculus*	Common Kestrel	1^a^
Passeriformes	Laniidae	*Lanius isabellinus*	Isabelline Shrike	2
	Pycnonotidae	*Pycnonotus cafer*	Red‐vented Bulbul	1
		*Pycnonotus leucogenys*	Himalayan Bulbul	1
	Hirundinidae	*Riparia riparia*	Sand Martin	1
	Phylloscopidae	*Phylloscopus trochilus*	Willow Warbler	1
		*Phylloscopus collybita*	Common Chiffchaff	9
	Acrocephalidae	*Acrocephalus stentoreus*	Clamorous Reed Warbler	19
		*Acrocephalus agricola*	Paddyfield Warbler	1
		*Acrocephalus scirpaceus*	Common Reed Warbler	3
	Sturnidae	*Acridotheres tristis*	Common Myna	1
	Muscicapidae	*Erithacus rubecula*	European Robin	1
		*Luscinia svecica*	Bluethroat	13
	Passeridae	*Passer domesticus*	House Sparrow	19
		*Passer hispaniolensis*	Spanish Sparrow	15
	Estrildidae	*Euodice malabarica*	Indian Silverbill	1

^a^ Sequence quality was lower.

### 3.1 Agreement Between Genetic and Phenotypic Species Identifications

#### 3.1.1 Species Where Genetic and Phenotypic Data Agreed

Genetic and phenotypic species identity matched for all but six cases, detailed in the following section (see also Table S1). Species identity was correctly returned by the automatic BOLD ID engine for 15 species. Automatic detection failed (with search result “A species level match could not be made”), but identification was readily found by examining the phylogenetic tree given by the ID engine, for an additional 14 species. Automatic detection failed for the following reasons. In six cases, the genetic and phenotypic species assignments agreed but there were discrepancies in the taxonomic naming of the individuals already in the BOLD database (e.g., a mix of individuals with and without subspecies‐level identifications, or a mix of different taxonomic concepts). For an additional six cases, our specimen's species was not assigned because the BOLD tree contained one or a few other specimens that are likely incorrectly labeled in the database (generally apparent mistakes between species with similar appearances). We reported these inconsistencies to the BOLD support staff, so our results may not be repeatable (pending corrections to the database). For two cases ( *Himantopus himantopus* and *Circus aeruginosus* ), the Qatari sequence clustered appropriately with a monophyletic group with the correct species, but the BOLD ID engine indicated multiple possible species identifications, likely because interspecific divergences in these genera are quite shallow.

In two cases, we had made misidentifications in the field, which we corrected after examining the field data in light of the genetic data. Specifically, one individual identified as *Phylloscopus trochilus* in the field was genetically assigned to be a *Phylloscopus collybita* ; these species are difficult to distinguish in the hand, and *Phylloscopus collybita* were also captured at the site, so this misidentification appears routine. Another individual identified as *Acrocephalus scirpaceus* in the field was genetically assigned as *Acrocephalus agricola* . These two species also have similar overall appearances, and unfortunately, the diagnostic wing measurements were not taken in the field. However, this individual had a shorter wing chord (54.5 mm) and bill length (7.7 mm) than the genetically confirmed *A. scirpaceus* that we captured (64.5 ± 0.87 mm; 8.7 ± 0.32 mm; *n* = 3). Because *A. agricola* has a shorter wing than *A. scirpaceus* (Svensson 1992), and occurs in Qatar as a rare vagrant (Gavin Farnell, pers. com, 2020), we determined that this individual was *A. agricola* .

In one case, *Bubo ascalaphus* , no previous sequence data were available for the species. It was therefore not possible to evaluate whether genetic and phenotypic assignment agreed.

#### 3.1.2 Discrepancies Between Genetic and Phenotypic Assignments

For four of the species we captured ( *Falco tinnunculus* , *Larus fuscus* , *Larus cachinnans* , and *Streptopelia decaocto* , each represented by a single individual), currently‐recognized species likely cannot be distinguished via sequencing at a single genetic locus. For each of these specimens, the BOLD phylogenies returned by our queries showed substantial non‐monophyly among samples from the field‐assigned species of the specimen and closely related species. Thus, while the samples clustered with the correct species, DNA barcodes did not successfully separate these particular species.

Finally, one *Spilopelia senegalensis* was a clear genetic match to *Spilopelia chinensis* . This individual had plumage relatively typical of *S. senegalensis* (i.e., without the spotted collar of *S. chinensis* ¸ Figure S3), and *S. chinensis* is not known to naturally occur in Qatar, so we retained our original field determination of species.

#### 3.1.3 Degree of Intra‐ Versus Interspecific Genetic Divergence

The barcoding gap was generally apparent in our data. That is, the within‐species distances were 0.31% ± 0.0% (0.00%–3.04%; mean ± SE, range; *n* = 88 individuals, 593 comparisons within 8 taxa) when excluding the possibly introgressed *Spilopelia senegalensis* sample (0.36 ± 0.00, 0.00%–4.90% when including it, *n* = 89 individuals, 601 comparisons within 8 taxa). Between‐species, within‐genus differences were 5.49% ± 0.01% (0.18%–16.68%, 71 individuals and 375 comparisons using 5 species) when all comparisons are included. Genus *Larus* has a complex mitochondrial history (Johnsen et al. 2010; Aliabadian et al. 2013); when we exclude comparisons involving the two *Larus* individuals, between‐species, within‐genus differences were 5.51% ± 0.01% (2.25%–16.68%, *n* = 69 individuals, 374 comparisons using 4 species). Between‐genus, within‐family distances were 12.55% ± 0.06% (9.50%–14.36%, 28 individuals, 24 comparisons using 8 species).

### 3.2 Ecology and Evolution of Qatari Avifauna

Most individuals, except where noted otherwise, did not have substantial molt or fat deposits when captured. Six of the *Phylloscopus collybita* showed substantial body molt. Molt was also observed in the body or tail of two of the *Acrocephalus stenoreus* (captured February 2020), one *Acridotheres tristis* (December 2019), on the head of the *Curruca* sp. (January 2020) and in the throats of two *Luscinia svecica* males (February 2020).

#### 3.2.1 Repeated Captures

Eight individuals were recaptured (and immediately released after inspection for body fat) at the Irkhaya pond area, out of the total of 45 birds ringed there between 24 January and 13 March 2020 (Table 2). Mist nets were mostly placed in two locations separated by approximately 350 m (one location used on all dates; the other used only from 31 January to 13 March). We did not make detailed behavioral observations about the territoriality of these individuals, nor keep systematic records of which net individuals were captured in (though the *Erithacus rubecula* and the male *Luscinia svecica* were recaptured in the same net). It seems plausible that these two individuals were territorial, and we stopped using these capture locations to diminish impact on the individuals due to repeated trapping.

**TABLE 2:** Summary of recaptures of marked individuals at Irkhaya ponds. Age and sex are indicated where this was known (2K indicates second calendar year; 3K+ is third calendar year or older).

Species	Date first caught	Date(s) recaptured	Ring number	Sex	Age
*Acrocephalus stenoreus*	24‐Jan‐20	21‐Feb‐20	8P18106		
	7‐Feb‐20	14‐Feb‐20	8P18110		
	7‐Feb‐20	21‐Feb‐20	8P18108		
	7‐Feb‐20	21‐Feb‐20	8P18109		
*Erithacus rubecula*	21‐Feb‐20	13‐Mar‐20	EN25013		2K
*Luscinia svecica*	24‐Jan‐20	14‐Feb‐20, 13‐Mar‐20	EN25001	Male	3K+
	31‐Jan‐20	14‐Feb‐20	EN25002	Female	3K+
	7‐Feb‐20	14‐Feb‐20	EN25003	Female	2K

#### 3.2.2 Genetic Diversity Within Species

Intraspecific distances among individuals sampled in Qatar (Table 3) averaged < 1% for most species. *Phylloscopus collybita* captured in Qatar belonged to both of the major clades in this group on the BOLD species tree. *Luscinia svecica* samples from Qatar similarly occurred throughout the BOLD species tree.

**TABLE 3:** Genetic distance (Kimura 2 parameter) among individuals of the same species captured in Qatar.

Species	*N*	Minimum	Mean	Maximum	SE
*Spilopelia senegalensis*	8^a^	0	0.20	0.74	0.01
*Lanius isabellinus*	2	0.5	0.50	0.50	0
*Phylloscopus collybita*	9	0	1.61	3.04	0.03
*Acrocephalus stentoreus*	19	0	0.42	1.46	0
*Acrocephalus scirpaceus*	3	0	0.20	0.30	0.05
*Luscinia svecica*	13	0	0.27	0.51	0
*Passer domesticus*	19	0	0.09	0.45	0
*Passer hispaniolensis*	15	0	0.07	0.36	0

^a^ The individual with COI sequence clustering with *Spilopelia chinensis* was excluded from this analysis.

In contrast, though with a limited sample size, all three Qatari‐captured *Acrocephalus scirpaceus* were in the eastern clade (with samples from Russia, Japan, Israel, Cyprus, and Djibouti; though many specimens do not have locations associated). The other clade in this tree was primarily represented by samples from Western Europe, Russia, and Burkina Faso (as well as one Israeli capture).

*Passer domesticus* samples from Qatar also were not widely dispersed across the tree for this species. Rather, they clustered in a relatively diverged subclade that largely consisted of birds from Iraq, Afghanistan, Kuwait, and Pakistan (16 of 19 sequences) or appeared as fairly basal in the tree (3 sequences). Previous work on a different mitochondrial gene (the control region) in *Passer domesticus* showed no sequence divergence among subspecies (Sætre et al. 2012), so it is unclear how the structure we observe in COI relates to subspecies status and broader evolutionary patterns.

#### 3.2.3 *Luscinia svecica*

In *Luscinia svecica*, some subspecies can be distinguished by size and variation in the color of the patch in the center of the throat (Zink et al. 2003; Johnsen et al. 2006), particularly for males. We captured one red‐spotted male, one white‐spotted male, and six individuals with a blended red and white spot similar to the hybrid subspecies *volgae*. Three were females with drab throats that could not be linked to a subspecies. Two males were molting their throat patches, making determination somewhat difficult. We captured more male than female *Luscinia svecica* (10 males vs. three females), and more young birds than old (10 birds in their second calendar year and three in at least their third calendar year), though our sample size is not sufficient for statistical analyses. The male *Luscinia svecica* captured repeatedly (Table 2) had no visible body fat in the first capture, but on the final capture had an intermediate fat score with fat deposits in the furcula and belly.

#### 3.2.4 Genus *Passer*

*Passer domesticus* and *Passer hispaniolensis* individuals largely appeared to be in breeding condition when we caught them. At one of the Irkhaya Farm sites, we captured mostly *Passer hispaniolensis* but also several *Passer domesticus* males (females are difficult to distinguish morphologically). Males of both species at this site were in breeding condition, based on cloacal palpation, and females had brood patches. We did not confirm the exact status of nests because these were inaccessible in thorn bushes on very steep slopes (Figure S2). Female *Passer domesticus* at a different field site visited 1–2 weeks later also had brood patches ( *Passer hispaniolensis* were not observed at that site). All individuals' COI sequence agreed with species assignment. This included 14 *Passer hispaniolensis* males and one female (where species assignment is less certain) and 16 *Passer domesticus* males, as well as one female and two immatures captured in locations where we only observed *Passer domesticus*.

## 4 Discussion

Our results highlight the value of using morphology and DNA barcoding together in initial surveys of avifauna in a relatively unexplored location. Overall, we found that the genetic and phenotypic species identifications matched well; we contributed sequence data for a species that was not previously represented in the database; and we gained initial insights into the evolutionary histories of species of interest.

### 4.1 Agreement Between Genetic and Phenotypic Species Identifications

The genetic sequence data showed generally low intraspecific divergences, indicating an absence of cryptic species within our sample. Species identifications made in the field and with the automatic BOLD ID engine largely matched, further supporting an absence of cryptic species. Difficulties in automatic species assignment using DNA barcodes were primarily due to complications with changing taxonomy, complex evolutionary histories of some species, and apparent labeling errors or misidentifications in the database. Database complications due to mixed taxonomic concepts have recently been highlighted by other authors (Pulgarín‐R et al. 2021). Errors in public databases have also been recently highlighted and can be relatively common (van den Burg and Vieites 2023). Notably, the BOLD database is actively curated and therefore may have fewer errors than less‐curated databases such as GenBank (Barreira et al. 2016). Though in a few instances such problems could represent a challenge for a naïve user of the database, they are generally straightforward for people with some taxonomic experience.

In contrast to the majority of cases, four of our 33 successfully‐sequenced species (12%) belonged to clades where DNA barcoding is insufficient to assign species due to complex biological histories. This is in line with previous work: Bilgin et al. (2016) found that seven of the 33 (21%) common European bird species they sampled could not be identified to species level due to complex evolutionary histories, although other papers report difficulties in less than 10% of species (Johnsen et al. 2010; Barreira et al. 2016). The genus *Larus*, which includes two of our four problematic species, has previously been noted as being difficult for identifications via DNA barcoding (Johnsen et al. 2010; Aliabadian et al. 2013).

### 4.2 Ecology and Evolution of Qatari Avifauna

#### 4.2.1 Migratory Connectivity

Migratory connectivity describes the extent to which separate breeding populations also live separately in the non‐breeding season, and it can sometimes be inferred from COI sequence (although mitochondrial markers with higher intraspecific variation may be more powerful; Lovette et al. 2004). *Acrocephalus scirpaceus* breeding in Qatar appears to show substantial migratory connectivity, that is, individuals overwintering in Qatar belong to only one genetic sub‐clade, where the genetic division corresponds to a migratory divide between breeding populations (Procházka et al. 2018). In contrast, two species, *Luscinia svecica* and *Phylloscopus collybita*, appeared to show low migratory connectivity. For these species, individuals sampled in this study spanned most of the observed diversity in COI sequences, so to the extent that COI diversity reflects breeding location, our samples were from diverse breeding locations. For *Luscinia svecica*, we could further assign subspecies based on throat patch color, and found that multiple subspecies occur in the same field site in Qatar. It is unclear how commonly different *Luscinia svecica* subspecies overwinter together, because most work on migration in this species has used methods that do not allow precise localization of overwintering areas (Arizaga and Tamayo 2013; Arizaga et al. 2015; Lislevand et al. 2015). However, subspecies *volgae* and *pallidogularis* both commonly migrate through the same site in Israel (Markovets and Yosef 2005). Understanding how a species' genetic diversity is geographically distributed during migration and in overwintering periods is important in working towards conserving these species (Vickery et al. 2014).

#### 4.2.2 Overwintering Ecology

We found an overabundance of male *Luscinia svecica* (10 vs. three females) and an overabundance of young birds (10 in second calendar year vs. three in at least the third). Similarly, an overabundance of males was reported in Morrocco (Musseau and Beslic 2017) and in Israel (Markovets and Yosef 2005). Markovets and Yosef (2005) speculate that males overwinter farther north than females, affording them a shorter migration distance to return to their northerly breeding ranges.

Repeated captures of individual *Luscinia svecica* and *Erithacus rubecula* suggest that these overwintering individuals may be territorial while in Qatar. *Luscinia svecica* is known to be territorial on wintering grounds in Portugal (Eybert et al. 1989). Wintering and/or stop‐over site fidelity is substantial in several of the species captured in this study ( *Luscinia svecica* , *Acrocephalus scirpaceus, Erithacus rubecula*, and, to a lesser extent, *Phylloscopus collybita* ; Pearson 1972; Cuadrado 1992; Cantos and Tellería 1994; Catry et al. 2003; Markovets and Yosef 2005). A combination of territoriality and site fidelity indicates that habitat changes on the wintering grounds could have a substantial impact on individual birds.

#### 4.2.3 Hybridization

We present tentative evidence of introgression between *Spilopelia senegalensis* and *Spilopelia chinensis*. To our knowledge, no wild hybrids between these two species are previously known, though hybrids and back crosses have previously been produced in captivity (e.g., Irwin 1959). Although *Spilopelia chinensis* is not native to Qatar, it is sold alive at the bird market in Doha, Qatar (ERAC pers. obs. on 25 February 2023; Figure S3), such that our observation might be linked to feralization. Understanding whether, and to what extent, interbreeding between these lineages is occurring in the wild in Qatar could shed light on reproductive barriers between the species.

Conversely, we found no evidence of hybridization between *Passer domesticus* and *Passer hispaniolensis* in Qatar, though these two species hybridize in some localities (Summers‐Smith and Vernon 1972; Summers‐Smith 1988). Differences in microhabitat or the timing of breeding are hypothesized to cause reproductive barriers in sympatry in some populations (Summers‐Smith and Vernon 1972; Summers‐Smith 1988), but in our study sites, the species were breeding at the same time and sometimes in the same microhabitats. Our data represent only a coarse test for hybridization, on a relatively small sample size of individuals, but our results may suggest that other reproductive barriers than microhabitat and chronology are effective in this population.

## 5 Conclusions

Even though a few studies have investigated bird populations in Qatar (e.g., Zogaris and Kallimanis 2016; Draidia et al. 2022) or other parts of the Arabian peninsula (e.g., Campbell et al. 2019), this region is under‐represented in the ornithological literature and in DNA barcode databases. Our study provides novel genetic information about Qatari avifauna and lays a foundation for studying species' ecology, particularly for migratory species on the less‐studied wintering grounds. Such knowledge may become particularly important for conservation, since there can be rapid changes in land use that potentially dramatically impact how birds use the region.

## Author Contributions

**Emily Rebecca Alison Cramer:** conceptualization (equal), formal analysis (equal), investigation (equal), writing – original draft (equal), writing – review and editing (equal). **Kuei‐Chiu Chen:** conceptualization (equal), investigation (equal), resources (equal), writing – review and editing (equal). **Arild Johnsen:** conceptualization (equal), resources (equal), writing – review and editing (equal).

## Conflicts of Interest

The authors declare no conflicts of interest.

## Supporting information


Figures S1–S3

Table S1

## Data Availability

Sequence and image data are available in the dataset DS‐QAVES on the Barcode of Life Database, BOLD: DOI: https://doi.org/10.5883/DS‐QAVES; sequence data are also on GenBank BankIt2972696: PV835474–PV835587. Data on the *Falco tinnunculus* sample is included in the public data for the QAVES project on BOLD (QAVES058‐21), but not in the dataset, as low sequence quality resulted in apparent stop codons. Morphological measurements are available at Data Dryad: https://doi.org/10.5061/dryad.kh18932kh.
