# Supplementary material for: Overwintering and Resident Birds in Qatar: Explorations With DNA Barcoding
Source: Ecol Evol. 2025 Jul 15;15(7):e71817. doi: 10.1002/ece3.71817 (PMC12263196; doi:10.1002/ece3.71817)
Supplement: Supplementary file 1 — Figures S1–S3 [file ECE3-15-e71817-s001.docx]

**Supplementary materials for “Overwintering and resident birds in Qatar: explorations with DNA barcoding” (ERA Cramer, KC Chen, A Johnsen)**


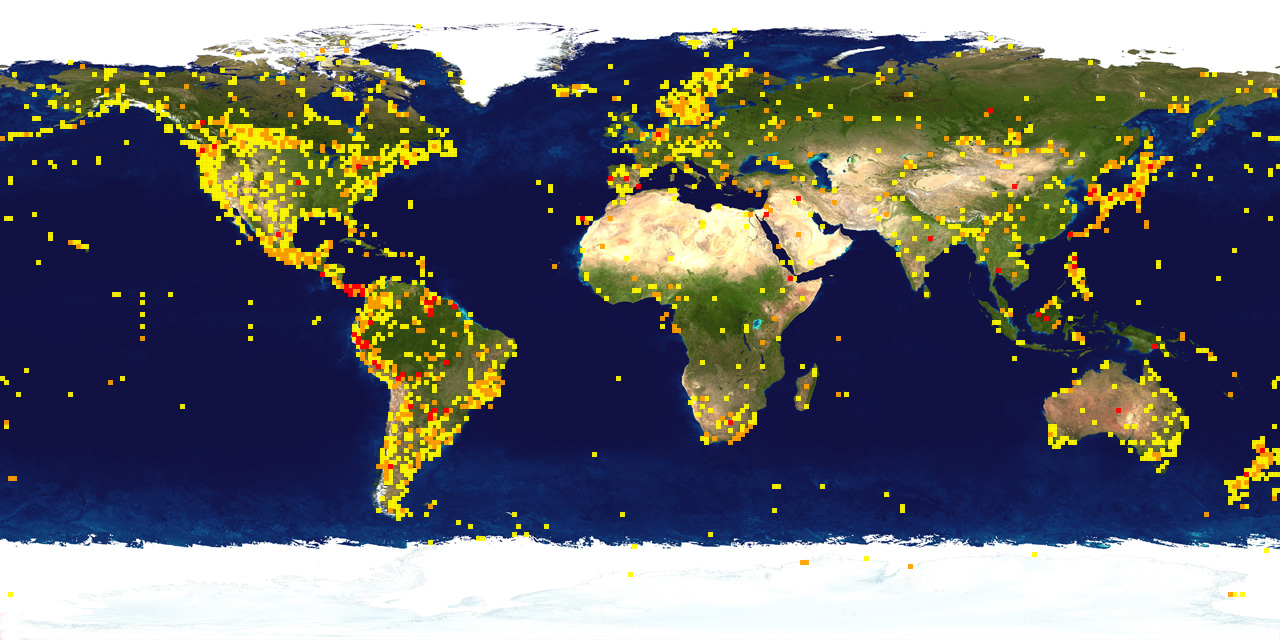


**Figure S1:**

**Map showing specimen occurrence data from 50,189 public avian records on BOLD.**

The color of the point indicates number of specimens, scaling from 1 (yellow) to 10,000 (red). (source: BoldSystems.org; search term “Aves”, date 12 December 2023).

*Description of field sites* (underlined words reflects label on the map, Figure 1; images of some sites in Figure S2)*:*

- Education City (25°18'58.0"N 51°26'20.8"E) is a semi-urban landscape with large expanses of lawn interspersed with dense hedges and trees (true trees and date palms, *Phoenix dactylifera*). Nets were placed within landscaped areas where shade conditions permitted. We captured birds on 15 Feb 2019, 2-3 October 2019, and 4 Dec 2019.
- Irkhaya is a government-owned farm producing feed for livestock. On its west side (25°00'44.3"N 51°09'30.5"E), wastewater filled two ponds that were surrounded by reeds (*Phragmites australis*), and reeds also grew in a relatively narrow band outside the dykes that create the pond structures. We visited this site most often because of the ease of access and because capture success was relatively high. On the north and east sides of the farm (25°00'32.9"N, 51°12'37.3"E), we observed relatively dense breeding populations of house and Spanish sparrows (breeding in desert thorn trees *Lycium shawii* and apparently feeding primarily on the nearby fields). Netting was performed on nine Fridays between 24 January 2020 - 13 March 2020.
- Al Karaana (24°59'07.6"N 51°02'27.7"E) is a set of ponds containing treated sewage effluent, which have recently been refurbished to remove historically dumped industrial and untreated waste (Draidia et al. 2022). At the time of fieldwork, the habitat was rapidly changing as vegetation (primarily reeds) began to grow around the edges of the newly re-formed ponds. Netting was conducted 19 Nov 2022. Because it is a popular location for local falconers (who often leave remains from prey birds in the desert) a number of the specimens sampled dead were from this area.
- Bin Ghannam Island is at the intersection of desert, wastewater runoff from a treatment plant, and mangrove (*Avicennia marina*) habitats, with some dense reeds and some more open habitat in close proximity. Nets were placed in both the reeds and in the open habitat (25°42'07.7"N 51°33'04.6"E), on 20 March 2020.
- Musfur (25°10'30.5"N 51°12'42.4"E) is a natural dry sinkhole that supports a large number of breeding *Passer domesticus*. The surrounding habitat is an open scrubland, without obvious large additions of water to the desert landscape. However, livestock are common in the area, with some nearby farms appearing to have feeding and watering stations for livestock. Birds were captured by placing mist nets across the sinkhole opening on 18 March 2020.


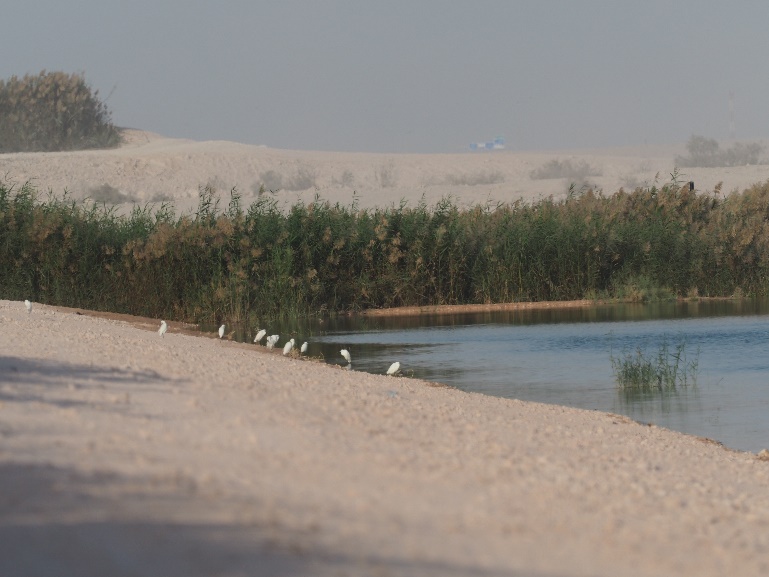
*
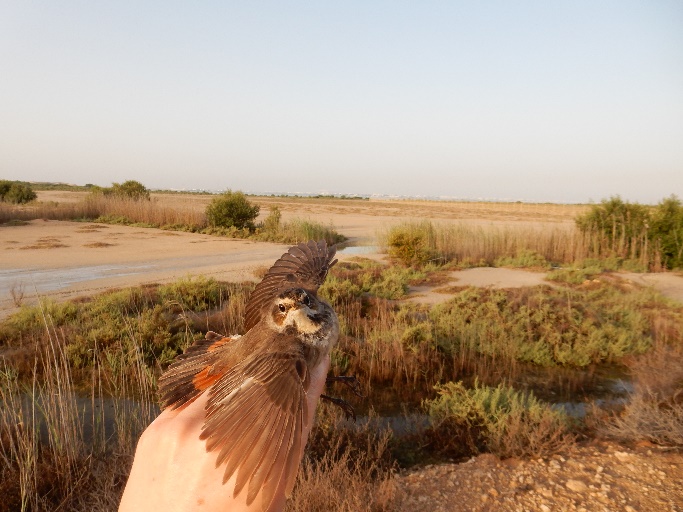
*
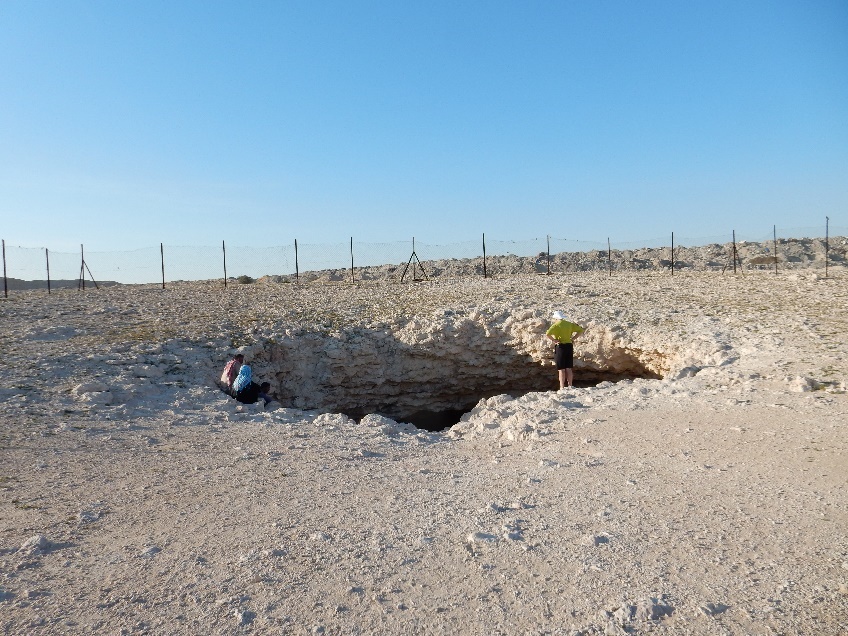

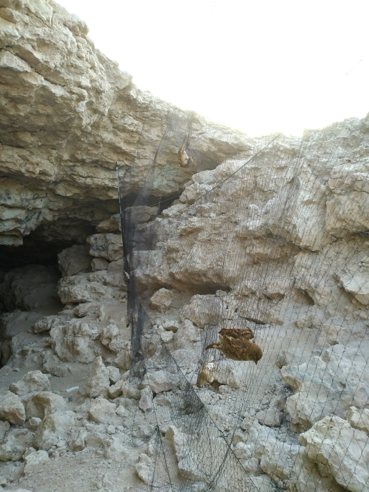

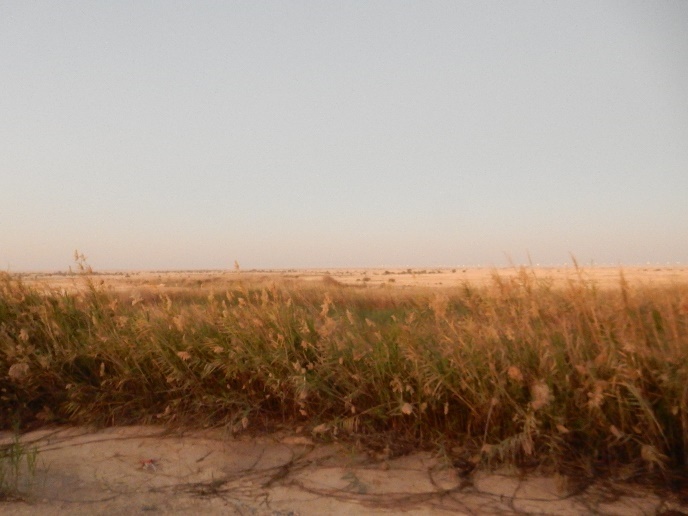

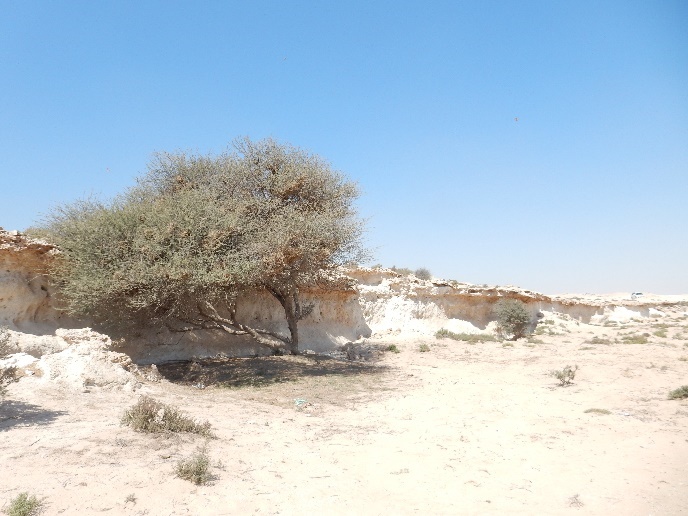


**Figure S2:**

**Images of field sites.**

Top: Musfur sinkhole (left, from the outside; right image showing positioning of the mist net). Middle left: Irkhaya pond reedbeds. Middle right: Site far to the east of Irkhaya farm, where *Passer hispaniolensis* and *Passer domesticus* were captured. Bottom left: Bin Ghannam Island. Bottom Right: Al Karaana Ponds.

*Measurement details*

Morphological measurements were:

- tarsus length: taken with dial calipers accurate to 0.1 mm, from the notch of the intertarsal joint to the distal end of the tarsus, with toes bent down at a 90 degree angle
- wing chord (not flattened): taken with a standard wing ruler to the nearest mm
- tail length: taken to the nearest mm by inserting a clear ruler between the central rectrices until it touched the bird’s body. The length of the longest tail feather was noted.
- bill length: taken with dial calipers to the nearest 0.1 mm, from the bill tip to the distal point of the nares
- bill depth and bill width: taken with dial calipers to the nearest 0.1 mm, at the distal point of the nares
- body mass: taken with a spring scale, with accuracy depending on the scale’s weight range
- age: for *Erithacus rubecula* and *Luscinia svecica,* age was determined by examining the edges of the primary coverts (Svensson 1992).


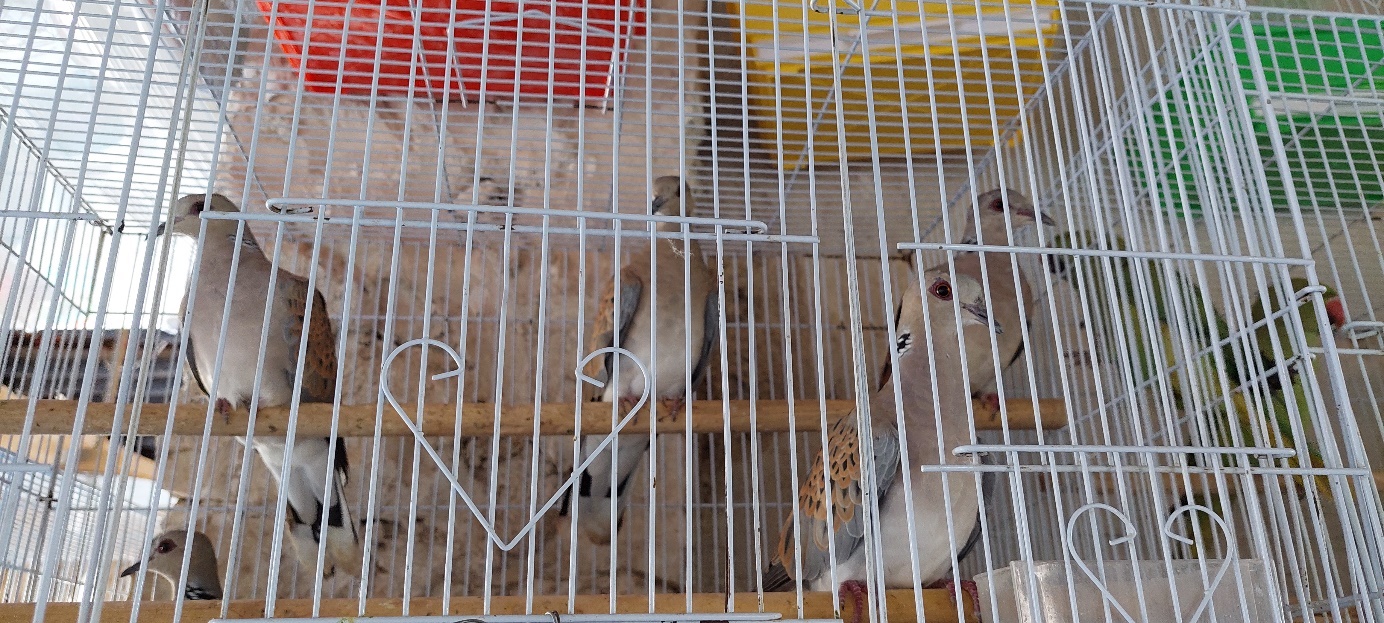

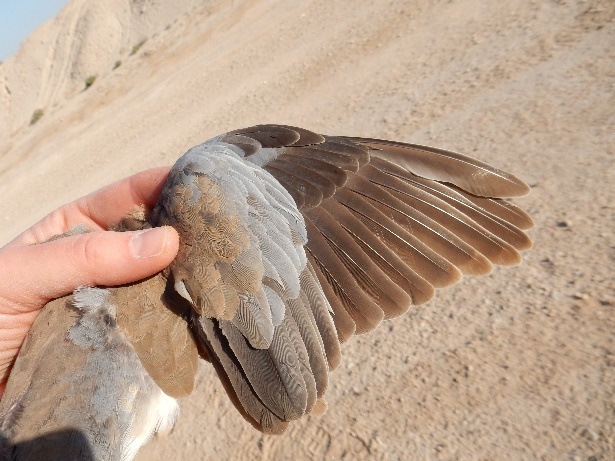

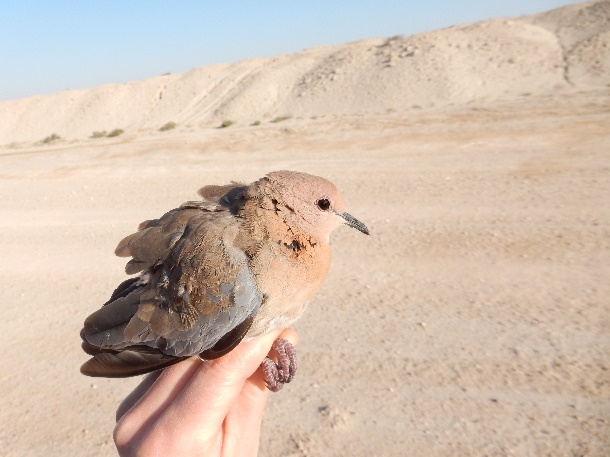


**Figure S3:**

**Spilopelia images.**

Top, *Spilopelia chinensis* in the Souq Waqif bird market in Doha, Qatar, 25 February 2023. Bottom: two views of the *Spilopelia senegalensis* (NHMO-BI- 106532 or QAVES038-21), which clustered with *S. chinensis* in COI sequence.
